# Differential contributions of choline phosphotransferases CPT1 and CEPT1 to the biosynthesis of choline phospholipids

**DOI:** 10.1016/j.jlr.2021.100100

**Published:** 2021-07-29

**Authors:** Yasuhiro Horibata, Hiroyuki Sugimoto

**Affiliations:** Department of Biochemistry, Dokkyo Medical University School of Medicine, Mibu, Tochigi, Japan

**Keywords:** phospholipids, phosphatidylcholine, phospholipid biosynthesis, phospholipid metabolism, choline phosphotransferase 1, choline/ethanolamine phosphotransferase 1, Kennedy pathway, *trans*-Golgi network, PUFA, radiolabeling, BFA, brefeldin A, CEPT1, choline/ethanolamine phosphotransferase 1, CHO-K1, Chinese hamster ovary-K1, CPT1, choline phosphotransferase 1, CRISPR, clustered regularly interspaced short palindromic repeats, DAG, 1,2-diacyl-*sn*-glycerol, DKO, double-KO, EPT1, ethanolamine phosphotransferase 1, HA, hemagglutinin, HEK293, human embryonic kidney 293, PC, phosphatidylcholine, PE, phosphatidylethanolamine, PL, phospholipid, plasmanyl-PC, 1-alkyl-2-acyl-*sn*-glycerophosphocholine, plasmenyl-PC, 1-alkenyl-2-acyl-*sn*-glycerophosphocholine, plasmenyl-PE, 1-alkenyl-2-acyl-*sn*-glycerophosphoethanolamine, TGN, *trans*-Golgi network

## Abstract

Choline phospholipids (PLs) such as phosphatidylcholine (PC) and 1-alkyl-2-acyl-*sn*-glycerophosphocholine are important components for cell membranes and also serve as a source of several lipid mediators. These lipids are biosynthesized in mammals in the final step of the CDP-choline pathway by the choline phosphotransferases choline phosphotransferase 1 (CPT1) and choline/ethanolamine phosphotransferase 1 (CEPT1). However, the contributions of these enzymes to the de novo biosynthesis of lipids remain unknown. Here, we established and characterized CPT1- and CEPT1-deficient human embryonic kidney 293 cells. Immunohistochemical analyses revealed that CPT1 localizes to the *trans*-Golgi network and CEPT1 to the endoplasmic reticulum. Enzyme assays and metabolic labeling with radiolabeled choline demonstrated that loss of CEPT1 dramatically decreases choline PL biosynthesis. Quantitative PCR and reintroduction of CPT1 and CEPT1 revealed that the specific activity of CEPT1 was much higher than that of CPT1. LC-MS/MS analysis of newly synthesized lipid molecular species from deuterium-labeled choline also showed that these enzymes have similar preference for the synthesis of PC molecular species, but that CPT1 had higher preference for 1-alkyl-2-acyl-*sn*-glycerophosphocholine with PUFA than did CEPT1. The endogenous level of PC was not reduced by the loss of these enzymes. However, several 1-alkyl-2-acyl-*sn*-glycerophosphocholine molecular species were reduced in CPT1-deficient cells and increased in CEPT1-deficient cells when cultured in 0.1% FBS medium. These results suggest that CEPT1 accounts for most choline PL biosynthesis activity, and that both enzymes are responsible for the production of different lipid molecular species in distinct organelles.

Phosphatidylcholine (PC) is the most abundant phospholipid (PL) in eukaryotic cell membranes, generally constituting approximately 40–50% of total cellular PL (1). There are two pathways for the biosynthesis of PC in mammalian cells: the phosphatidylethanolamine (PE) methylation pathway, and the CDP-choline pathway (or Kennedy pathway) (2). In the PE methylation pathway, PE *N*-methyltransferase transfers a methyl group from *S*-adenosylmethionine to PE to generate PC (3). In mammals, approximately 30% of PC is generated by the PE methylation pathway in the liver, whereas the main pathway for PC biosynthesis in other tissues is the CDP-choline pathway (4).

PC is biosynthesized by the CDP-choline pathway in three reactions. The first step is the formation of phosphocholine by choline kinase (5). The second step is catalyzed by CTP:phosphocholine cytidylyltransferase, encoded by *PCYT1*, which activates phosphocholine by interaction with CTP to produce CDP-choline (6). PC is finally generated by transferring phosphocholine from CDP-choline to 1,2-diacyl-*sn*-glycerol (DAG), with the release of CMP by choline phosphotransferase (CPT). Two CPTs, choline phosphotransferase 1 (CPT1) (7) and choline/ethanolamine phosphotransferase 1 (CEPT1) (8), have been identified in mammals. CPT1 exclusively utilizes CDP-choline as a donor substrate to produce PC, whereas CEPT1 has dual specificity for both CDP-choline and CDP-ethanolamine as substrates to synthesize PC and PE, respectively. Kinetic analysis revealed that CEPT1 prefers CDP-choline over CDP-ethanolamine as the phosphobase donor (9, 10).

Another type of choline PL in mammalian cells is ether-linked lipids such as 1-alkyl-2-acyl-*sn*-glycerophosphocholine (plasmanyl-PC) and 1-alkenyl-2-acyl-*sn*-glycerophosphocholine (plasmenyl-PC). Plasmanyl-PC and plasmenyl-PC are synthesized by the CDP-choline pathway in which 1-alkyl-2-acyl-*sn*-glycerol and 1-alkenyl-2-acyl-*sn*-glycerol, respectively, are utilized as lipid acceptors instead of DAG. Plasmenyl-PC can also be synthesized by methylation of the ethanolamine in 1-alkenyl-2-acyl-*sn*-glycerophosphoethanolamine (plasmenyl-PE or plasmalogen) (11, 12, 13). It is widely accepted that plasmanyl-PC is distributed in most tissues, whereas plasmenyl-PC is distributed in limited organs such as the heart and skeletal muscle (14).

Both CPT1 and CEPT1 are integral membrane proteins with a CDP-alcohol phosphatidyltransferase catalytic motif, an amino acid sequence conserved in enzymes catalyzing the displacement of CMP from a CDP-alcohol. In addition to the donor substrate specificity mentioned previously, these enzymes have different properties. CEPT1 is universally expressed in mammalian organs, whereas CPT1 is most abundant in testis, followed by colon, small intestine, heart, prostate, and spleen. CEPT1 is localized to the endoplasmic reticulum (ER) and nuclear membranes in Chinese hamster ovary-K1 (CHO-K1) cells (15) and to the ER in human embryonic kidney 293 (HEK293) cells (16). In contrast, CPT1 is localized in the Golgi apparatus in CHO-K1 cells (15). These enzymes therefore synthesize choline PL at different subcellular sites. The in vitro lipid acceptor specificities of CPT1 (7) and CEPT1 (8) have already been studied. However, the detailed contribution of these enzymes to the de novo biosynthesis of choline PLs molecular species in mammalian cells remains unknown.

In the present study, we studied the subcellular localization of CPT1 and CEPT1 in HEK293 cells and compared choline PL biosynthesis in *CPT1*-KO, *CEPT1*-KO, and double-KO (DKO) cells. We found that CPT1 is localized to the *trans*-Golgi network (TGN) and CEPT1 to the ER. A deficiency of CEPT1 caused dramatic decreases in both in vitro CPT activity and de novo biosynthesis of choline PL from choline. We demonstrated that CEPT1 has much higher specific enzyme activity than does CPT1 for synthesis of the lipid using a quantitative PCR and reintroduction study. Profiling of the newly synthesized choline PL from deuterium-labeled choline revealed that these enzymes have a similar preference for the synthesis of PC molecular species but show a different preference for plasmanyl-PC molecular species with PUFA. Finally, we found that the endogenous levels of PC were not affected by the loss of these enzymes. In contrast, when cells were cultured in 0.1% FBS, the content of some plasmanyl-PC molecular species was significantly reduced in *CPT1*-KO cells and increased in *CEPT1*-KO cells. Taken together, our findings suggest for the first time that CEPT1 has higher activity than CPT1 for producing choline PL, and both enzymes are responsible for the production of different choline PL molecular species in distinct organelles.

## Materials and Methods

### Cell culture

HEK293 cells were cultured in DMEM (high glucose) with 10% FBS. Cells were grown on plastic petri dishes coated with 1% Cellmatrix Type I-C (Nitta Gelatin, Osaka, Japan) at 37°C in a humidified incubator containing 5% CO_2_. Alternatively, cells were cultured in HE100 medium (Gmep, Inc, Fukuoka, Japan), a medium suitable for culturing cells without serum. Briefly, cells were first cultured in HE100 medium containing 1% FBS for 1 day and then cultured in the medium containing 0.1% FBS for another 2 days. Medium was not changed to suppress cell detachment.

### Establishment of *CPT1*-KO, *CEPT1*-KO, and DKO HEK293 cells by genome editing using clustered regularly interspaced short palindromic repeats/Cas9

To generate *CPT1*-KO HEK293 cells using clustered regularly interspaced short palindromic repeats (CRISPR)/Cas9 technology, a DNA fragment encoding guide RNA of CPT1 (sense, 5ʹ-caccgGGGCCGGGTCCGCGCCGCGC-3ʹ; antisense, 5ʹ-aaacGCGCGGCGCGGACCCGGCCCc-3ʹ) was cloned into the pSpCas9(BB)-2A-Puro (pX459) vector (Addgene, Cambridge, MA). Cells were transfected with the vector using Lipofectamine 2000 transfection reagent (Thermo Fisher Scientific, Waltham, MA) according to the manufacturer's instructions. Forty-eight hours after transfection, the cells were cultured in the presence of 1 μg/ml puromycin for 2 days, and then, the surviving cells were plated as single colonies in 96-well plates. *CEPT1*-KO cells were generated as reported previously (16). To establish DKO cells, we prepared a pSpCas9(BB)-2A-Neo vector, which has a neomycin resistance gene instead of puromycin, and then a DNA fragment encoding the guide RNA of CEPT1 (sense, 5ʹ-caccgTGAGTGGGCATCGATCAACA-3ʹ; antisense, 5ʹ-aacTGTTGATCGATGCCCACTCAc-3ʹ) was cloned into the vector. *CPT1*-KO cells were transfected with the vector containing the guide RNA of CEPT1 as described previously. After culturing in the presence of 800 μg/ml geneticin (Thermo Fisher Scientific) for 10 days, the surviving cells were plated onto 96-well plates to obtain single clones. To verify mutations, genomic DNA was isolated from the cells using an Easy DNA Extraction Kit (Kaneka Corp., Tokyo, Japan), and the DNA fragments around the guide RNA target were amplified by PCR using Go Taq DNA polymerase (Promega, Madison, WI). The primers used were: 5ʹ-ACCGGTGAGTCCAGCCCGGCAGTCGCAGG-3ʹ/5ʹ-TAGTGGCGTGACAGTCTCCAGGCGGCCCAG-3ʹ for CPT1 and 5ʹ-CCTCCTTTTCTGGTCCTGTTTGATACTTAC-3ʹ/5ʹ-TATCTTTAGTTAAAATGACCCCACGATCCC-3ʹ for CEPT1. After purifying using a QIAquick gel extraction kit (Qiagen, Hilden, Germany), the PCR products were cloned into T-vector pMD20 (Takara Bio, Inc., Shiga, Japan). The DNA sequences of several clones were determined using an ABI PRISM 377-XL DNA sequencer (Applied Biosystems, Foster City, CA) and a BigDye Terminator v1.1 Cycle Sequencing Kit (Applied Biosystems).

### Immunocytochemistry

Open reading frames of CPT1 and CEPT1 fused with a hemagglutinin (HA) tag at the C terminus were amplified by PCR and cloned into the pCAG vector (Wako Pure Chemical Industries, Osaka, Japan). Cells were grown on glass coverslips coated with 1% Cellmatrix Type I-C. Expression vectors were transfected into the cells using Lipofectamine 2000. To label the ER, the cells were cotransfected with pDsRed-ER vector encoding a red fluorescent protein fused with the ER retention sequence of calreticulin (Takara Bio, Inc.). After 24 h, the cells were fixed with 4% paraformaldehyde in PBS for 15 min, washed with PBS, permeabilized with 0.1% Triton X-100 (w/v) for 10 min, and then blocked with 5% skim milk (w/v) for 30 min. The cells were then incubated with anti-HA (Cell Signaling Technology, Danvers, MA), anti-*cis*-Golgi matrix protein 130 kDa (GM130) (Sigma-Aldrich, St. Louis, MO), and anti-TGN, 46 kDa (TGN46) (Proteintech, Chicago, IL) antibodies overnight at 4°C, followed by washing and immunostaining with fluorescently labeled secondary antibodies conjugated with Alexa Fluor 488 or 594 (Thermo Fisher Scientific) for 1 h at room temperature. Nuclei were stained with 4′,6-diamidino-2-phenylindole, and the samples were observed with a confocal microscope (LSM780; Zeiss, Oberkochen, Germany).

### In vitro CPT assay

CPT activity was measured as described previously (7). Briefly, cells were lysed in 50 mM Tris-HCl buffer, pH 8.0, with an ultrasonic bath sonicator for 10 s, and the amount of protein in the lysate was measured using a BCA protein assay kit (Thermo Fisher Scientific). The assay reaction mixture (30 μl) contained 50 mM Tris-HCl buffer, pH 8.0, 20 mM MgCl_2_, 1 mM EDTA, 0.003% Tween-20 (w/v), cell lysate (5 μg of protein), 20 μM CDP-choline [1,2-^14^C], and 1 mM 18:1 DAG (Sigma-Aldrich). After incubation at 37°C for the time indicated, the reaction was stopped by adding 300 μl of chloroform-methanol (1:1, v/v), and then 110 μl of water was added. After centrifugation at 12,000 *g* for 5 min, the organic phase was applied to a TLC plate (Merck, Darmstadt, Germany), which was developed with chloroform-methanol-water (65:25:4, v/v/v). Radiolabeled PC was analyzed using a FLA-7000 imaging analyzer and quantified using ImageQuant TL, version 8.1 (GE Healthcare UK Ltd, Amersham, UK).

### Metabolic labeling of choline PL with radiolabeled choline

Before metabolic labeling, the cells were washed once with PBS and cultured in 10% FBS containing DMEM without choline chloride for an hour, and then the cells were cultured in the same medium containing 0.2 μCi/ml of ^14^C-choline chloride [1,2-^14^C] (American Radiolabeled Chemicals, St. Louis, MO) for the time indicated. After washing with PBS twice, the cells were lysed in distilled water. Lipid was extracted from the cells using the Bligh and Dyer method (17). Radiolabeled choline PL was analyzed by TLC and quantified as described previously. Radioactivity in the cell lysate was quantified using an ALOKA LSC-6100 scintillation counter (Hitachi, Ltd, Tokyo, Japan) to evaluate choline uptake. The results were normalized against the amount of cellular protein.

### Cell proliferation assay

Cell proliferation was assayed using Cell Counting Kit-8 (Dojindo, Ltd, Kumamoto, Japan) according to the manufacturer's instructions. Briefly, cells were plated into 96-well plates and cultured for 2 days. After adding the Cell Counting Kit-8 solution (tetrazolium) to each well, plates were incubated at 1 h. Formazan dye generated by NADH dehydrogenases in cells was quantified by measuring absorbance at 450 nm using a microplate reader.

### Absolute quantification of mRNA copy number by real-time PCR

Total RNA was isolated from cells using a RNeasy mini kit (Qiagen) and then reverse transcribed using ReverTra Ace qPCR RT Master Mix (TOYOBO, Osaka, Japan) according to the manufacturer's instructions. Quantitative PCR was carried out on a 7300 Real-Time PCR System (Applied Biosystems) using FastStart Universal SYBR Green Master (Roche Diagnostics, Mannheim, Germany). The primers used were 5ʹ-GCATGTTGAGATTTGGAAAAGTGG-3ʹ/5ʹ-TCCACCTAGAAATCCAAGAACTGG-3ʹ for CPT1 and 5ʹ-CAGTGATTGGAGGACCACCT-3ʹ/5ʹ-AGGACACTTGTTCCTGCTATTGT-3ʹ for CEPT1. The PCR amplicon was cloned into T-vector pMD20 and used for calculating the copy number.

### Reintroduction of CPT1 and CEPT1 into DKO cells and immunoblotting

The expression vectors of HA-tagged CPT1 or HA-tagged CEPT1 described previously were transfected into DKO cells using Lipofectamine 2000. After 24 h, the cells were lysed in 20 mM Tris-HCl buffer, pH 8.0, containing 0.2% Triton X-100 (w/v) and proteinase inhibitor cocktail (Nacalai Tesque, Kyoto, Japan). Proteins were separated with SDS-PAGE, transferred to PVDF membranes (FluoroTrans; Pall Corp., Port Washington, NY) using a Trans-Blot SD Semi-Dry Transfer blotter (Bio-Rad Laboratories, Hercules, CA), and then the membranes were incubated with 5% (w/v) skim milk in TBS for 1 h. The membranes were then incubated with anti-HA tag (Cell Signaling Technology) and anti-GAPDH (Wako Pure Chemical Industries) antibodies overnight at 4°C, washed three times with TBS containing 0.1% Tween-20 (w/v), and then incubated with horseradish peroxidase-conjugated IgGs for 1 h at room temperature. The membranes were washed three times with TBS containing 0.1% Tween-20 and stained with Clarity Western ECL Substrate (Bio-Rad) according to the manufacturer's instructions and visualized using a ChemiDoc Touch imaging system (Bio-Rad). Protein density was quantified using Image Lab software (Bio-Rad). Results were normalized against the density of GAPDH.

### Metabolic labeling of choline PL with deuterium-labeled choline and quantification of lipid molecular species by LC-MS/MS

After culturing the cells for 1 h in choline-deficient DMEM with 10% FBS, 1 mM *d9*-choline chloride (trimethyl-*d9*) (Cambridge Isotope Laboratories, Andover, MA) was added to the medium. Brefeldin A (BFA) treatment was performed at a concentration of 20 μg/ml for 2 h as described (16). After culturing for the time indicated, the cells were washed with PBS twice and lysed in distilled water. Lipid was extracted with methanol containing 0.1 nmol of internal standards (*d70*-PC; Olbracht Serdary Research Laboratories, Toronto, Canada). To eliminate plasmenyl-PC, lipid samples were treated with 0.15 N HCl at room temperature for 2 h as described (18). Choline PL molecular species were analyzed by reverse-phase HPLC using an L-column 2 ODS column (3 μm, 2.0 × 100 mm) (Chemicals Evaluation and Research Institute, Tokyo, Japan) coupled to a 5500 QTRAP mass spectrometer (Sciex, Inc., Framingham, MA). A binary gradient consisting of solvent A (acetonitrile:methanol:water, 1:1:3, v/v/v, containing 5 mM ammonium acetate) and solvent B (2-propanol containing 5 mM ammonium acetate) was used. The gradient profile was 0–1 min, 95% A; 1–9 min, 5–95% B linear gradient; and 9–13 min, 95% B. The flow rate was 0.2 ml/min, and the column temperature was 40°C. Endogenous choline PL was detected in multiple reaction monitoring mode by selecting the *m/z* of the PL molecular species at Q1 and the precursor ion of *m/z* 184 at Q3 in positive ion mode. For deuterium-labeled choline PL synthesized via the CDP-choline pathway and PE methylation pathway, the precursor ion of *m/z* 193 (*d9*-labeled choline) and 187 (*d*3-labeled choline) at Q3 was monitored, respectively. Lipids were quantified using MultiQuant version 2.0 (Sciex) and normalized against the internal standards and amount of protein.

### Statistical analysis

Quantitative data are presented as means ± SD. Statistical significance was assessed using the Student's *t*-test or one-way ANOVA with Dunnett's post hoc test. A *P* value of <0.05 was considered statistically significant.

## Results

### CPT1 is distributed in the TGN, whereas CEPT1 is localized in the ER

**Fig. 1:**
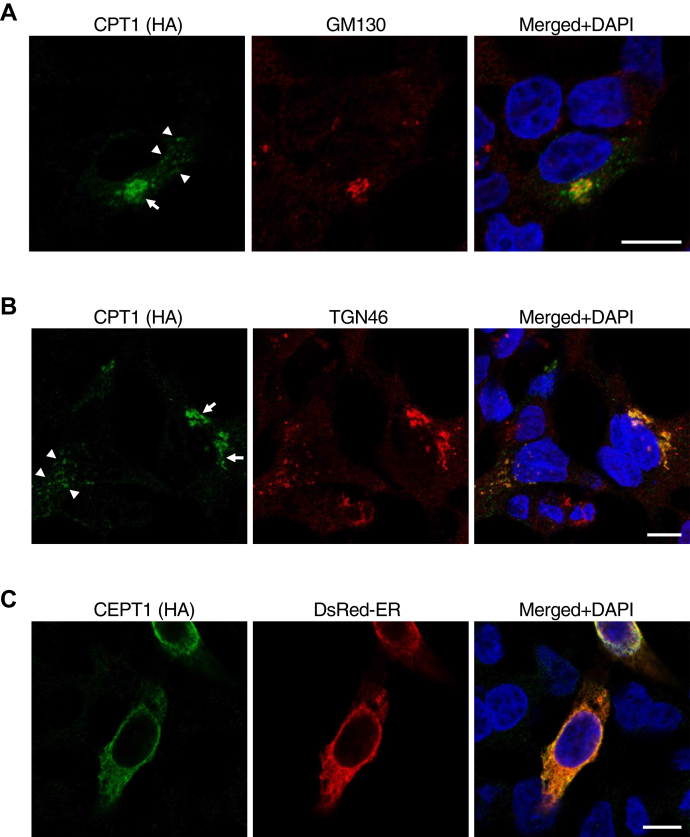
Immunocytochemistry of CPT1 and CEPT1 in HEK293 cells. HEK293 cells were transfected with the expression vectors for CPT1 (A, B) or CEPT1 (C) tagged with HA at the C terminus. After fixation, the cells were permeabilized with 0.1% Triton X-100 (w/v), immunostained with anti-HA mouse antibody, then with anti-mouse IgG Alexa Fluor 488 (green). The *cis*- and *trans*-Golgi were stained with anti-GM130 and anti-TGN46 rabbit antibodies, respectively, followed by antirabbit IgG Alexa Fluor 594 (red). The ER was visualized using DsRed-ER (red). Nuclei were stained with DAPI (blue). Arrows indicate perinuclear Golgi-like structures. Arrowheads indicate dot-like structures. Bars indicate 10 μm.

CPT1 and CEPT1 are expressed in kidney, as determined previously (7, 8), and thus, we used HEK293 cells in this study. We compared the intracellular distribution of CPT1 and CEPT1 in the cells by transfecting the expression vectors encoding CPT1 or CEPT1 fused with an HA tag at the C terminus into HEK293 cells, then staining with anti-HA-tag antibody followed by the second antibody conjugated with Alexa Fluor 488. Previous study revealed that the intracellular distribution of CPT1 is to the Golgi apparatus in CHO-K1 cells (15). Consistent with this, here we observed that the green signal of CPT distributed to the perinuclear Golgi-like structure (shown by *arrows* in Fig. 1A, B). The signal of the perinuclear structure is partially merged with the red signals of GM130, a *cis*-Golgi marker (Fig. 1A). Interestingly, we found that the CPT1 signal also distributed in dot-like structures (arrowheads). Both the perinuclear and dot-like signals merged well with TGN46, a TGN marker (Fig. 1B). In contrast, the green signal of CEPT1 was colocalized with the red signal of DsRed-ER, a fluorescent protein distributed in the ER (Fig. 1C). These results suggest that the intracellular location for the biosynthesis of choline PL by CPT1 and CEPT1 is mainly the TGN and ER, respectively.

### The de novo biosynthesis of choline PL from ^14^C-choline in *CPT1*-KO, *CEPT1*-KO, and DKO cells

**Fig. 2:**
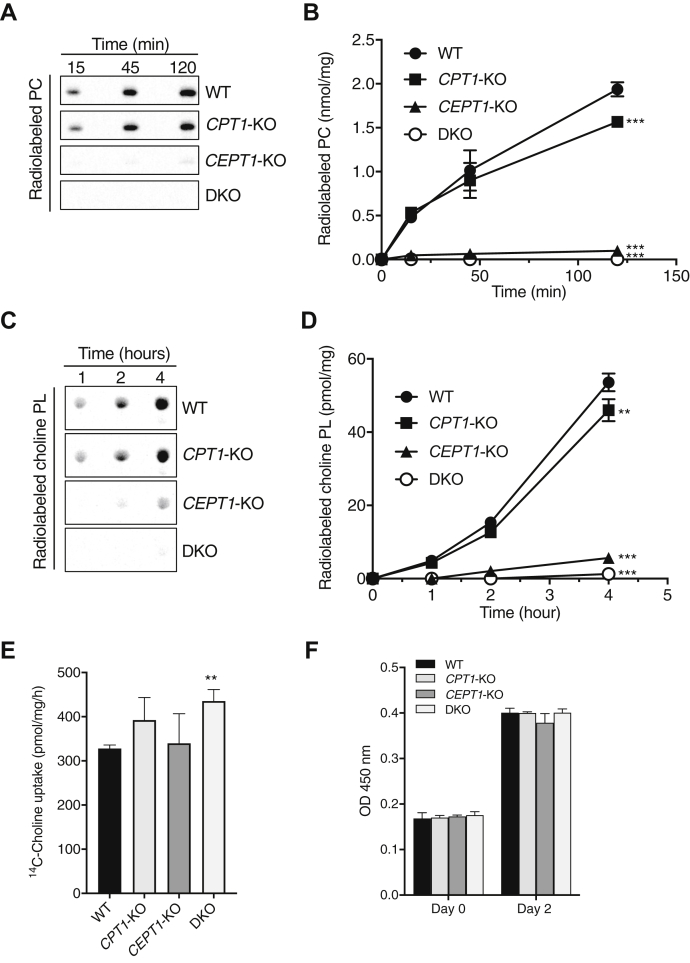
CPT activity and the de novo biosynthesis of choline PL from radiolabeled choline in WT, *CPT1*-KO, *CEPT1*-KO, and DKO cells. A and B: CPT activity in cell lysates from WT, *CPT1*-KO, *CEPT1*-KO, and DKO HEK293 cells. Enzymatic activity was measured using radiolabeled CDP-choline and 18:1 DAG as substrates at 37°C for the time indicated. The synthesized radioactive PC was analyzed by TLC (A) and quantified using ImageQuant TL, version 8.1 (B). Values shown are means ± SD from three independent assays. C and D: Metabolic labeling of choline PL with radiolabeled choline. Cells were incubated with ^14^C-choline for the time indicated. After extraction, radioactive choline PL was analyzed by TLC (C). The amount of radiolabeled lipid in (C) was quantified and normalized by the amount of total cellular protein (D). E: The total radioactivity in the cells was measured using a liquid scintillation counter. Data are means ± SD from three independent culture dishes. ∗∗ and ∗∗∗ indicate *P* < 0.01 and *P* < 0.001 as compared with WT, respectively, determined using one-way ANOVA with Dunnett's post hoc test. F: Cell proliferation was assessed using Cell Counting Kit-8. The amount of the formazan dye generated by NADH dehydrogenases in cells is quantified by measuring absorbance at 450 nm.

To evaluate the contributions of CPT1 and CEPT1 to choline PL biosynthesis, we established *CPT1*-KO and *CEPT1*-KO HEK293 cells using the CRISPR/Cas9 system. We also prepared DKO cells by introducing a CRISPR/Cas9 vector containing CEPT1 guide RNA into *CPT1*-KO cells (supplemental Fig. S1). CPT activity in these cells was assayed in vitro for the time indicated using radiolabeled CDP-choline as a phosphocholine donor and 18:1 DAG as a lipid acceptor. Radioactive PC produced was separated by TLC (Fig. 2A), and the radioactivity of the lipid was quantified (Fig. 2B). At a reaction time of 120 min, the levels of PC synthesized in WT, *CPT1*-KO, and *CEPT1*-KO cells were 1.94 ± 0.1, 1.56 ± 0.1, and 0.10 ± 0.1 nmol/mg, respectively, indicating that the majority of CPT activity in HEK293 cells is derived from CEPT1.

The de novo biosynthesis of choline PL was then analyzed by a metabolic labeling experiment using radiolabeled choline. After cells were cultured with ^14^C-choline for the time indicated, the radiolabeled choline PL was analyzed by TLC (Fig. 2C) and the radioactivity of the lipid was quantified (Fig. 2D). After labeling for 4 h, the levels of radiolabeled choline PL in WT, *CPT1*-KO, and *CEPT1*-KO cells were 53.5 ± 2.4, 46.0 ± 3.0, and 5.7 ± 0.1 nmol/mg, respectively. Figure 2E shows the uptake of radiolabeled choline into the cells. DKO cells showed slightly higher choline uptake than the other cells. Taken together, these results suggest that CEPT1 synthesizes about 90% of the choline PL in HEK293 cells.

The effect of CPT1 and/or CEPT1 deficiency on cell proliferation was assessed by NADH dehydrogenase-based method. As shown in Fig. 2F, there was no significant difference between WT, *CPT1*-KO, *CEPT1*-KO, and DKO cells, suggesting that these enzymes are not critical for cell proliferation.

### The mRNA level of CPT1 and CEPT1 in HEK293 cells

**Fig. 3:**
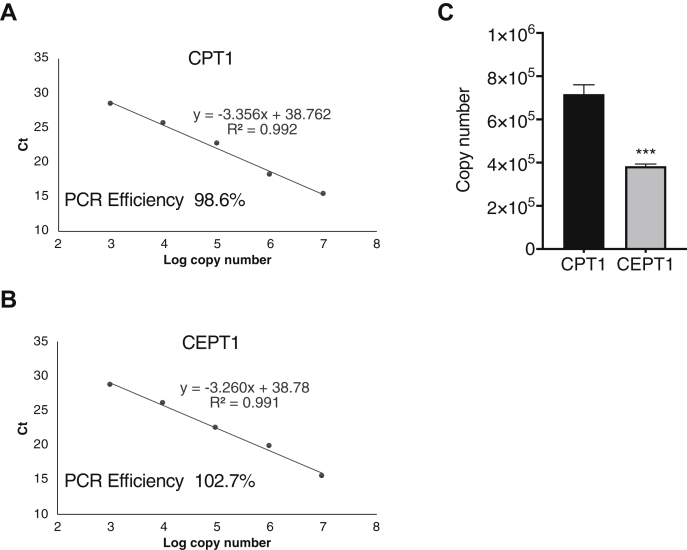
Copy numbers of CPT1 and CEPT1 transcripts in HEK293 cells. Calculation of the amplification efficiency of CPT1 (A) and CEPT1 (B) in PCR experiments. Serial dilutions of vector containing each target were amplified by real-time PCR. Ct values and logarithmic scales are plotted. C: Real-time PCR was performed using complementary DNA synthesized using total RNA derived from HEK293 cells as template, and the copy numbers of CPT1 and CEPT1 transcripts were calculated. Data are means ± SD from three independent culture dishes. ∗∗∗ indicates *P* < 0.001 as compared with CPT1 determined using the Student's *t*-test.

In vitro enzymatic assay and metabolic labeling experiments using radiolabeled choline suggested that the activity of CPT1 for choline PL biosynthesis is much less than that of CEPT1 in HEK293 cells. One possible reason is that the expression level of CPT1 is lower than that of CEPT1. To examine this possibility, transcript copy numbers were monitored by absolute quantification by real-time PCR. As shown in Fig. 3A, B, the PCR results were validated by calculating the amplification efficiency. After synthesizing complementary DNA using total RNA derived from HEK293 cells, quantitative PCR was performed to calculate the copy number. As shown in Fig. 3C, the copy number of CPT1 and CEPT1 was about 7.2 ± 0.4 × 10^5^ and 3.8 ± 0.1 × 10^5^, respectively, indicating that the mRNA level of CPT1 is about twice as high as that of CEPT1. These results suggest that the lower activity of CPT1 for choline PL biosynthesis is not because of its lower expression compared with CEPT1.

### CPT activity and de novo choline PL biosynthesis in CPT1- or CEPT1-reintroduced DKO cells

**Fig. 4:**
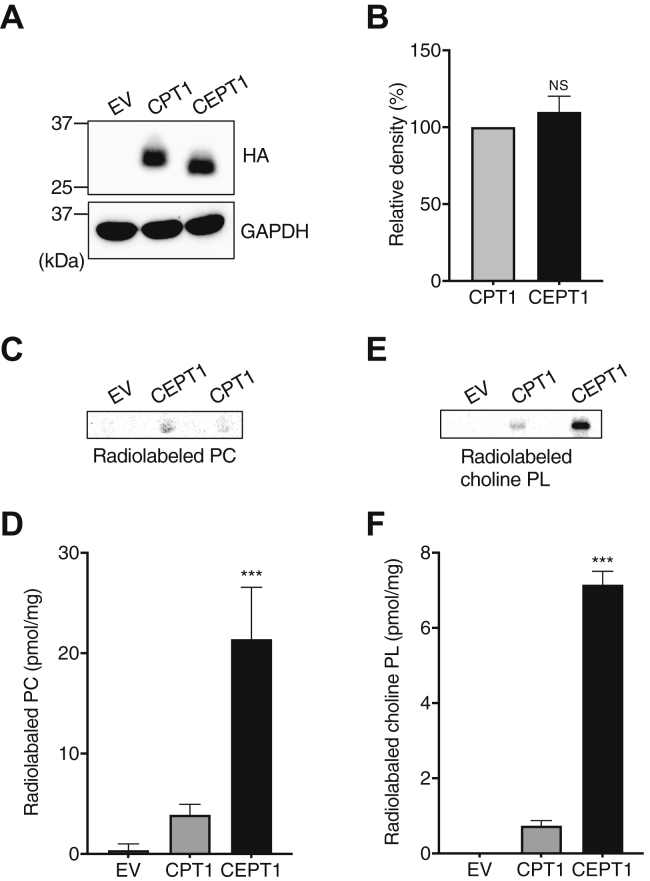
CPT activity and the de novo biosynthesis of choline PL in CPT1- or CEPT1-reintroduced DKO cells. A, B: DKO cells were transfected with empty vector (EV) or with vector containing CPT1 or CEPT1 fused with a HA tag at the C terminus. After 24 h, the cell lysates were separated by SDS-PAGE and HA-tagged proteins were analyzed by immunoblotting using anti-HA antibody. GAPDH was used as a protein loading control (A). The relative band density of HA-tagged protein was measured. Data were normalized by GAPDH (B). C, D: CPT activity was measured using the lysates of DKO cells transfected with EV or with vector containing CPT1 or CEPT1. After 120 min of reaction with radiolabeled CDP-choline, radioactive PC was analyzed by TLC (C) and quantified (D). E, F: Metabolic labeling of choline PL with radiolabeled choline. DKO cells were transfected with EV or with vector containing CPT1 or CEPT1. After 24 h, the cells were incubated with ^14^C-choline for 2 h. After extraction, the radioactive lipids were analyzed by TLC (E) and quantified (F). The amount of radiolabeled lipid was normalized by the amount of total cellular protein. ∗∗∗ indicates *P* < 0.001 as compared with CPT1 determined using the Student's *t*-test.

Despite its higher expression level, CPT1 contributes less to choline PL synthesis than does CEPT1 in HEK293 cells. A possible explanation is that CPT1 has lower specific enzyme activity as a CPT per protein compared with CEPT1. To verify this hypothesis, expression vectors containing HA-tagged CPT1 or CEPT1 were reintroduced into DKO cells. As shown in Fig. 4A, 33- and 30-kDa protein bands were stained by anti HA-tag antibody after the reintroduction of CPT1 and CEPT1 into DKO cells, respectively. We also quantified the density of these protein bands and confirmed that the protein levels of HA-tagged CPT1 and CEPT1 were almost equal (Fig. 4B). In vitro CPT activity was then assayed using the cell lysate described previously. As shown in Fig. 4C, D, the levels of ^14^C-PC synthesized in the CPT1- and CEPT1-reintroduced cells were 3.9 ± 1.0 and 21.4 ± 5.2 nmol/mg, respectively, demonstrating that the specific activity of CEPT1 is about five times as high as that of CPT1. Next, we performed metabolic labeling experiments with ^14^C-choline. As shown in Fig. 4E, F, the levels of radiolabeled choline PL in the CPT1- and CEPT1-reintroduced DKO cells were 0.74 ± 0.1 and 7.2 ± 0.4 nmol/mg, respectively, indicating that the activity of CEPT1 for choline PL synthesis was about 10 times higher than that of CPT1. Taken together, these results suggest that the lower activity of CPT1 is due to its lower specific enzyme activity compared with that of CEPT1.

### Comparison of newly synthesized choline PL molecular species from deuterium-labeled choline

**Fig. 5:**
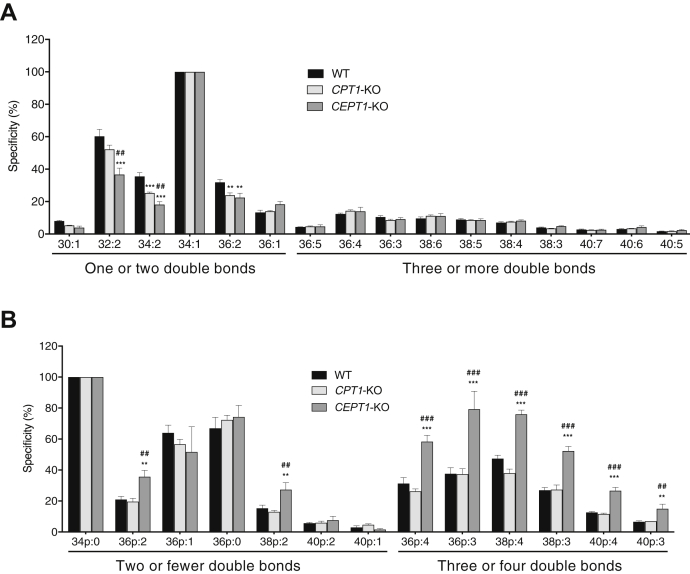
Specificity for the de novo biosynthesis of choline PL molecular species by CPT1 and CEPT1. WT, *CPT1*-KO, or *CEPT1*-KO cells were incubated with *d9*-labeled choline for 2 h. After extraction, the levels of *d9*-labeled PC and plasmanyl-PC molecular species were quantified using LC-MS/MS. The relative values of each PC and plasmanyl-PC molecular species to 34:1 (A) and 34p:0 (B), respectively, were calculated. Data are means ± SD from three independent culture dishes. ∗∗ and ∗∗∗ indicate *P* < 0.01 and *P* < 0.001, respectively, as compared with WT. ## and ### indicates *P* < 0.01 and *P* < 0.001, respectively, as compared with *CPT1*-KO. Statistical significance was assessed using one-way ANOVA with Dunnett's post hoc test.

Next, we analyzed the preference of CPT1 and CEPT1 for the de novo biosynthesis of choline PL molecular species. Cells were cultured with *d9*-choline for 2 h, and then the lipids were extracted and analyzed by LC-MS/MS. Choline PL newly biosynthesized from *d9*-choline can be distinguished from unlabeled endogenous lipids because of a 9-Da mass difference. Consistent with our radiolabeling results, the level of *d9*-labeled PC molecular species in *CEPT1*-KO cells was much lower than that of WT and *CPT1*-KO cells (supplemental Fig. S2A). To compare the preference of CEPT1 and CPT1 for the synthesis of PC molecular species, the relative values of each PC molecular species compared with 34:1 were calculated. Figure 5*A* shows that both *CPT1*-KO cells and *CEPT1*-KO cells showed a similar preference: that is, both enzymes favored the generation of PC molecular species in the order 34:1 > 32:2 > 34:2 = 36:2. Similar results were obtained using CPT1- or CEPT1-reintroduced DKO cells (supplemental Fig. S3A).

Next, we monitored ether-linked choline PL. Mammalian cells contain two types of ether choline PLs: plasmenyl-PC and plasmanyl-PC. The molecular weights of these lipids with the same fatty acids differ by 2 Da. This makes it difficult to distinguish between plasmanyl-PC and plasmenyl-PC by their molecular weight because a double bond in unsaturated fatty acids also causes a 2 Da difference. An alkenyl group in plasmenyl-PC is known to be degraded by acid treatment, whereas plasmanyl-PC is stable in acidic conditions (19). We therefore removed plasmenyl-PC by treating the lipid samples with HCl before LC-MS/MS analysis. As shown in supplemental Fig. S4A, B, we found that almost all the ether-linked choline PL in HEK293 cells is plasmanyl-PC because an almost identical plasmanyl-PC composition was observed both before and after HCl treatment. On the other hand, almost all the ether-linked ethanolamine PL is plasmenyl-PE because almost all the lipids were degraded by HCl treatment (supplemental Fig. S4C).

Similar to the results obtained for *d9*-labeled PC, the level of *d9*-labeled plasmanyl-PC molecular species in *CEPT1*-KO cells was much lower than that of WT and *CPT1*-KO cells (supplemental Fig. S2B), suggesting that plasmanyl-PC is primarily generated by CEPT1. The relative values of each plasmanyl-PC molecular species compared with 34p:0 were calculated (Fig. 5*B*). CPT1 and CEPT1 were found to show similar preference for the synthesis of plasmanyl-PC with two or fewer double bonds except 36p:2 and 38p:2. In contrast, the preference of CPT1 for plasmanyl-PC with PUFAs, such as 36p:4, 36p:3, 38p:4, 38p:3, 40p:4, and 40p:3, was higher than that of WT and CEPT1. We obtained similar results with CPT1- or CEPT1-reintroduced DKO cells that CPT1 has higher specificity for plasmanyl-PC with PUFA (supplemental Fig. S3B). These results suggest that CPT1 and CEPT1 have similar preference for the synthesis of PC molecular species but show different preferences for plasmanyl-PC molecular species with PUFA.

CPT1 and CEPT1 are localized in the TGN and ER, respectively (Fig. 1) and showed different preference for the generation of plasmanyl-PC molecular species. We investigated the possibility that this preference is due to their subcellular distribution by assessing the effect of BFA, a drug that promotes fusion of the Golgi membrane with the ER. BFA treatment redistributed CPT1 from TGN to the ER (supplemental Fig. S5A). As shown in supplemental Fig. S5B, C, the treatment did not significantly change the preference for plasmanyl-PC molecular species, suggesting that the observed preference is not because of the distinct distributions of CPT1 and CEPT1.

We also monitored choline PL biosynthesized by the PE methylation pathway by measuring *d3*-labeled PC and plasmanyl-PC molecular species by LC-MS/MS after metabolic labeling with *d9*-choline (4, 20). As shown in supplemental Fig. S6, the conversion of *d9*-choline into *d3*-labeled choline PL is very low (about 1% of *d9*-labeled choline PL). In addition, there were no differences in the synthesis of *d3*-labeled choline PL between WT, *CPT1*-KO, *CEPT1*-KO, and DKO cells, indicating that the PE methylation pathway remains unchanged by the loss of CPT1 and/or CEPT1.

### The endogenous levels of PC and plasmanyl-PC molecular species

We investigated the endogenous levels of choline PL in CPT1- and/or CEPT1-deficient cells. We confirmed that FBS contains non-negligible levels of PC, plasmanyl-PC, lyso-PC, and lyso-plasmanyl-PC (supplemental Fig. S7), and then minimized the supply of these lipids from FBS using HE100 medium, a chemically defined medium suitable for culturing HEK293 cells without serum. Because complete removal of FBS dramatically impaired cell adhesion to plates, we cultured cells in the presence of 0.1% FBS for 2 days without medium change to suppress cell detachment.

**Fig. 6:**
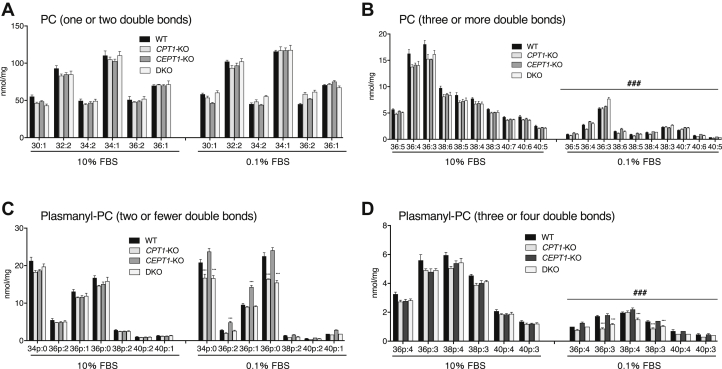
Amount of endogenous PC and plasmanyl-PC molecular species in WT, *CPT1*-KO, *CEPT1*-KO, and DKO cells cultured with 10% FBS or 0.1% FBS. Cells were cultured in HE100 medium containing 10% FBS or 0.1% FBS for 2 days. After lipids were extracted from the cells, the levels of PC with one or two double bonds (A), PC with three or more double bonds (B), plasmanyl-PC with two or fewer double bonds (C), and plasmanyl-PC with three or four double bonds (D) were quantified using LC-MS/MS. Lipid levels were normalized by the amount of total cellular protein. Data are means ± SD from three independent culture dishes. ∗∗∗ indicates *P* < 0.001 as compared with WT. ### indicates *P* < 0.001 as compared with 10% FBS. Statistical significance was determined using one-way ANOVA with Dunnett's post hoc test.

Figure 6A, B shows the levels of PC molecular species with one or two double bonds, and three or more double bonds, respectively. Even though DKO cells showed severe defects in PC biosynthesis from choline, no significant decrease in the endogenous levels of PC molecular species was observed. These results suggest that CPTs, CPT1 and CEPT1, are not essential for the maintenance of endogenous PC. Unexpectedly, the levels of PC molecular species with three or more double bonds were broadly lower in all cells cultured in 0.1% FBS (Fig. 6*B*). As shown in supplemental Fig. S8, it was found that the levels of DAG molecular species with arachidonic acid but not palmitoleic acid or oleic acid were significantly reduced in cells cultured in 0.1% FBS, suggesting that FBS is required for the supply of PUFA.

Figure 6C, D shows the levels of plasmanyl-PC molecular species with two or fewer double bonds, and three or four double bonds, respectively. There were no significant changes in plasmanyl-PC when the cells were cultured in 10% FBS-containing medium. In contrast, when the cells were cultured in 0.1% FBS, plasmanyl-PC molecular species, such as 34p:0, 36p:0, 36p:3, and 38p:3, were decreased in both *CPT1*-KO and DKO cells (Fig. 6*D*). These results suggest that CPT1 is important for the maintenance of several plasmanyl-PC molecular species under the serum-restricted conditions. Unexpectedly, plasmanyl-PC molecular species such as 36p:2 and 36p:1 was increased in *CEPT1*-KO cells (Fig. 6*C*). Similar to the results of PC, the endogenous levels of plasmanyl-PC with PUFA were widely reduced when cells were cultured in 0.1% FBS (Fig. 6*D*), supporting our idea that FBS supplies PUFA.

## Discussion

Mammalian cells contain the two distinct CPTs, CPT1 and CEPT1, which catalyze the final step of the CDP-choline pathway to produce choline PLs, such as PC, plasmanyl-PC, and plasmenyl-PC. The genes for these enzymes were identified over 20 years ago (7, 8), but the contributions of these enzymes to the de novo choline PL biosynthesis remain poorly understood. In this study, we established *CPT1*-KO and *CEPT1*-KO HEK293 cells using genome editing and compared choline PL biosynthesis by in vitro enzyme assays and metabolic labeling using isotopic choline. We found that loss of CEPT1 causes a dramatic decrease in the biosynthesis of both PC and plasmanyl-PC (Figs. 2 and supplemental Fig. S2). In contrast, a deficiency of CPT1 had less effect on choline PL synthesis even though its mRNA level is about double that of CEPT1 (Fig. 3), likely because CPT1 has lower specific enzyme activity as a CPT than does CEPT1 (Fig. 4). These results suggest for the first time that a major part of choline PL activity in the CDP-choline pathway is derived from CEPT1 in HEK293 cells.

Previous studies on the subcellular fractionation of rat liver demonstrated that CPT activity is distributed in both the ER and Golgi fractions (21, 22). Our immunocytochemistry results (Fig. 1) suggest that the enzymatic activities of the ER and Golgi fractions of liver are derived from CEPT1 and CPT1, respectively. In addition, the activity of CPT in the ER fraction is reportedly more than 10 times that of the Golgi fraction in liver tissue (22). Our data showing that CEPT1 has higher activity than CPT1 in HEK293 cells are consistent with the results obtained using rat tissue.

We compared the levels of PC and plasmanyl-PC molecular species newly synthesized from *d9*-labeled choline using LC-MS/MS and found that CPT1 and CEPT1 similarly favored the generation of PC with one or two double bonds in the order 34:1 > 32:2 > 34:2 = 36:2 (Figs. 5A and supplemental Fig. S3A). In contrast, CPT1 favored the synthesis of plasmanyl-PC with PUFA compared with CEPT1 (Figs. 5B and supplemental Fig. S3B). BFA treatment had no effect on this preference (supplemental Fig. S5), and thus, subcellular localization is apparently not important for the preference of the enzymes. These results suggest that CPT1 and CEPT1 synthesize distinct plasmanyl-PC molecular species.

Even though the de novo biosynthesis of choline PL is dramatically reduced in *CEPT1-KO* and DKO cells, no significant reduction of endogenous PC content was observed (Fig. 6A, B) when cells were cultured in 10% FBS. In contrast, the levels of PC molecular species with PUFA were widely decreased in all cells cultured in 0.1% FBS (Fig. 6B). These results suggest that FBS in medium supplies PC and/or lipid precursors for PC synthesis such as PUFA and lyso-PC. Similar to the results of PC, there was no significant difference in the levels of plasmanyl-PC when cells were cultured in 10% FBS (Fig. 6C, D). Although CEPT1 was much contributed to the synthesis of plasmanyl-PC than CPT1 (supplemental Fig. S2), significant decreases in the levels of lipid molecular species, such as 34p:0, 36p:0, 36p:3, and 38p:3, were observed in *CPT1*-KO and DKO cells cultured in 0.1% FBS (Fig. 6C, D). Unexpectedly, plasmanyl-PC molecular species such as 36p:2 and 36p:1 were increased in *CEPT1*-KO cells cultured in 0.1% FBS compared with WT. We cannot acceptably explain these contradictory results now. Choline PL levels are maintained by the reacylation and the remodeling pathways by lyso-PC acyltransferases (23) and phospholipases (24). Therefore, it is possible that the endogenous level of choline PL is not certainly reflected by the rate of lipid biosynthesis by CEPT1 and CPT1. Further research is required into the homeostasis and/or biosynthetic regulation of choline PL.

We previously studied the contribution of CEPT1 to the de novo biosynthesis of ethanolamine PL and demonstrated that this enzyme most favors the synthesis of PE 34:1 (16). Here, we found that CEPT1 most favors the generation of PC 34:1 (Figs. 5A and supplemental Fig. S3A). These results suggested that DAG 34:1 is the preferred lipid acceptor for the transfer of both CDP-choline and CDP-ethanolamine by CEPT1. We previously also analyzed the de novo biosynthesis of ethanolamine PL by ethanolamine phosphotransferase 1 (EPT1), an enzyme that specifically utilizes CDP-ethanolamine to synthesize ethanolamine PL and, like CPT1, is localized in the Golgi apparatus (25). Interestingly, we found that loss of EPT1, but not CEPT1, caused a significant decrease in the endogenous level of ether-linked plasmenyl-PE in HEK293 cells (16), HeLa cells, and human fibroblasts (18). The present study shows that the endogenous level of plasmanyl-PC is reduced by the loss of CPT1 (Fig. 6C, D), suggesting that Golgi-localizing CPT1 and EPT1 may be important for the maintenance of ether-linked PLs. Our present findings will facilitate further studies aimed at understanding the metabolism of lipids in the Golgi apparatus.

Functional differences between CPT1 and CEPT1 are not yet fully clear. In yeast, a consumption of DAG by CPT1 was reported to be required for the Sec14p-dependent regulation of Golgi-mediated protein transport (26). Here, we found that CPT1 is localized in the TGN, suggesting that this enzyme may also be involved in Golgi transport in mammalian cells. Muscle-specific *Cept1*-KO mice showed an increase in muscle insulin sensitivity after being fed a high-fat diet, suggesting that CPT1 cannot completely compensate for the loss of CEPT1 (27). The present study on the contributions of these enzymes to choline PL biosynthesis will help future investigations into the different biological importance of CPT1 and CEPT1.

In summary, here we demonstrated that CPT1 and CEPT1, the final enzymes for the synthesis of choline PL in the CDP-choline pathway, are mainly distributed in the TGN and ER, respectively. In HEK293 cells, most choline PL is synthesized by CEPT1 because the specific enzyme activity of CEPT1 is much higher than that of CPT1. Both enzymes have similar specificity for the synthesis of PC molecular species, but CPT1 has higher preference for the synthesis of plasmanyl-PC molecular species with PUFA than does CEPT1.

## Data availability

All data are contained in the article and supplemental data.

## Supplemental data

This article contains supplemental data.

## Conflict of interest

The authors declare that they have no conflicts of interest with the contents of this article.
