# Supplementary material for: Differential contributions of choline phosphotransferases CPT1 and CEPT1 to the biosynthesis of choline phospholipids
Source: J Lipid Res. 2021 Jul 29;62:100100. doi: 10.1016/j.jlr.2021.100100 (PMC8387743; doi:10.1016/j.jlr.2021.100100)
Supplement: Supplemental Figs. S1–S7 [file mmc1.pdf]

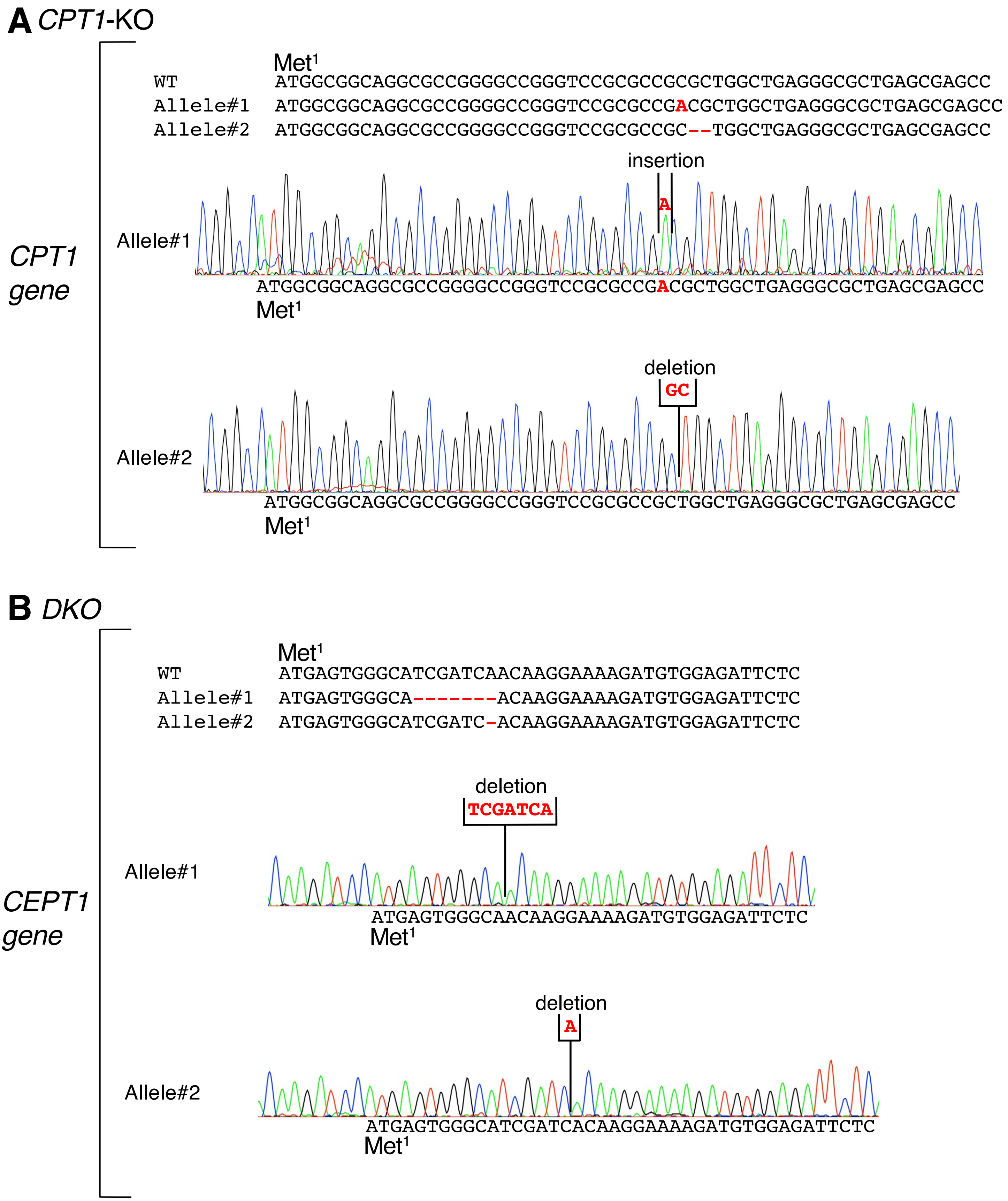

Supplemental Fig. S1. DNA sequences of CPT1 and CEPT1 genes in *CPT1*-KO and DKO cells. Alignment of genome sequences between WT and *CPT1*-KO (A) and DKO (B) cells. DKO cells were generated by introducing a vector containing CEPT1 guide RNA into *CPT1*-KO cells. The position of the first methionine is shown as Met^1. Deleted and inserted DNA sequences are shown by dashed lines and red bold, respectively. Waveform data are shown below the alignments. The DNA sequence of the CEPT1 gene in *CEPT1*-KO cells was described in our previous study (16).

Supplemental Figure S1

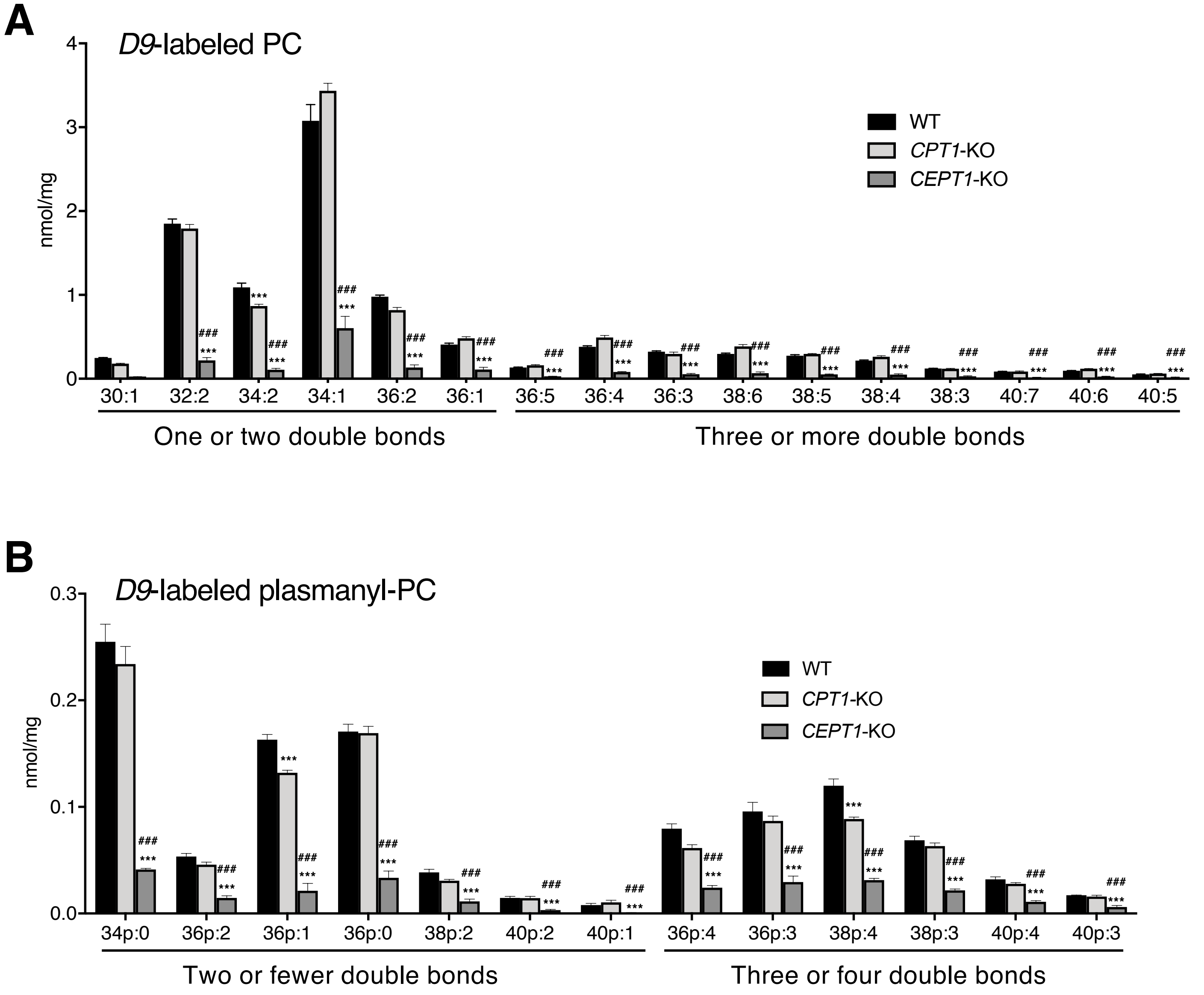

Supplemental Fig. S2. The *de novo* biosynthesis of PC and plasmanyl-PC from *d9*-labeled choline *via* the CDP-choline pathway.

WT, *CPT1*-KO, or *CEPT1*-KO cells were incubated with *d9*-labeled choline for 2 hours. After extraction, the levels of *d9*-labeled PC (A) and plasmanyl-PC (B) molecular species were quantified using LC-MS/MS. The lipid amount was normalized by the amount of total cellular proteins. Data are means ± S.D. from three independent culture dishes. *** indicates $P < 0.001$ as compared with WT. ### indicates $P < 0.001$ as compared with *CPT1*-KO. Statistical significance was assessed using one-way ANOVA with Dunnett's post hoc test.

Supplemental Figure S2

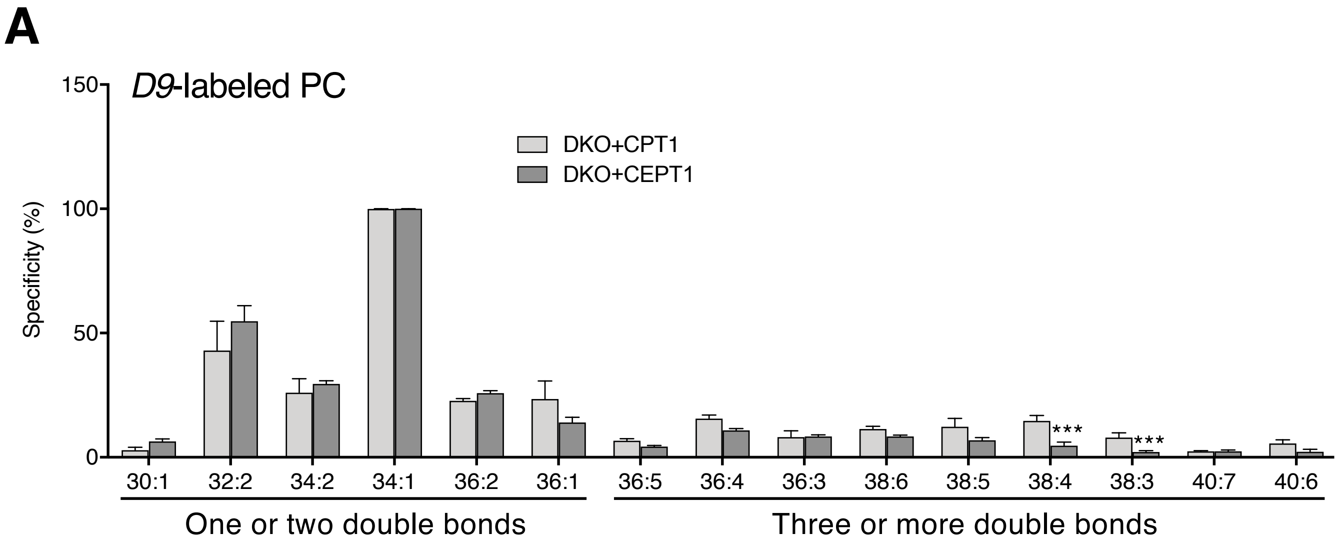

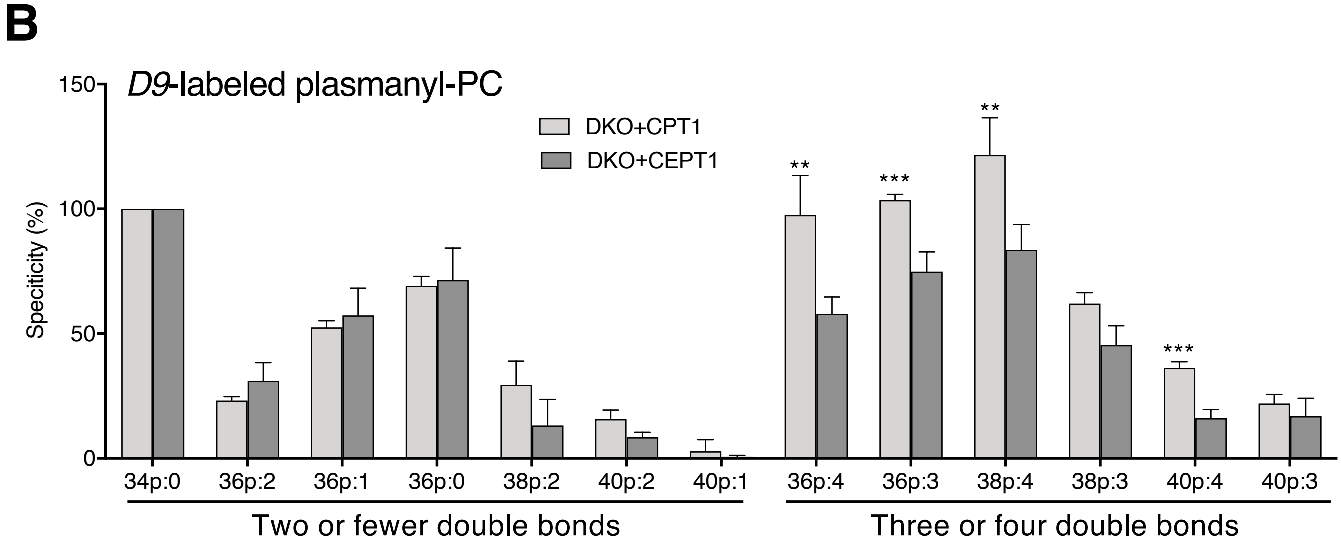

Supplemental Fig. S3. Specificity for the *de novo* biosynthesis of choline PL molecular species in CPT1- or CEPT1-reintroduced DKO cells.

DKO cells transfected with CPT1 (DKO+CPT1) or CEPT1 (DKO+CEPT1) were incubated with *d9*-labeled choline for 4 hours. After extraction, the levels of *d9*-labeled PC and plasmanyl-PC molecular species were quantified using LC-MS/MS. The relative values of each PC and plasmanyl-PC molecular species to 34:1 (A) and 34p:0 (B), respectively, were calculated. Data are means ± S.D. from three independent culture dishes. ** and *** indicates $P < 0.01$ and $P < 0.001$ as compared with DKO+CEPT1 determined using the Student's t-test.

Supplemental Figure S3

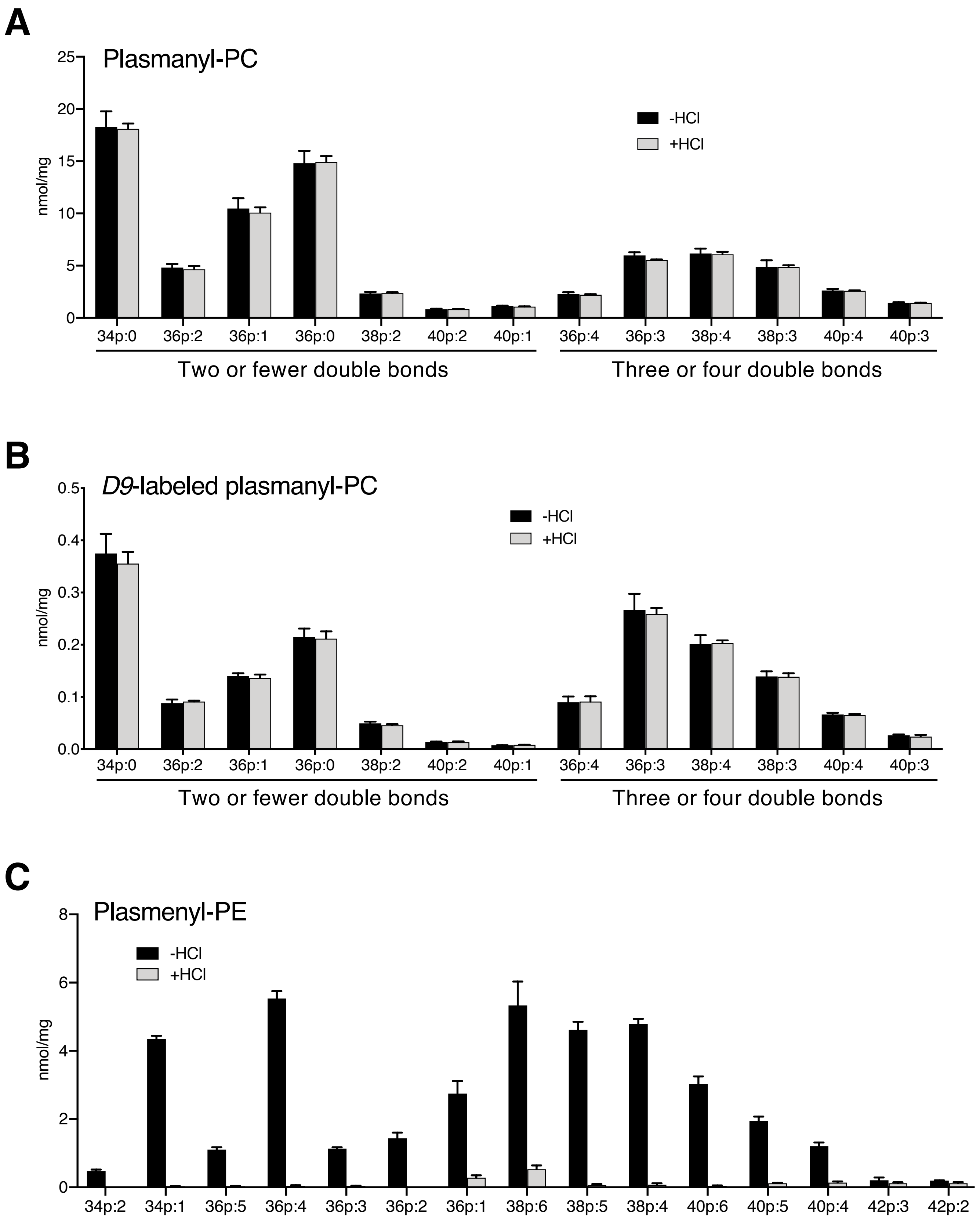

Supplemental Fig. S4. Treatment of lipid samples with HCl to remove PLs containing the alkenyl - group.

HEK293 cells were incubated with *d9*-labeled choline for 2 hours. After extraction, the lipids were treated with 0.15 N HCl at room temperature for 2 hours. The levels of plasmanyl-PC (A), *d9*-labeled plasmanyl-PC, and plasmenyl-PE molecular species (C) were quantified using LC-MS/MS.

Supplemental Figure S4

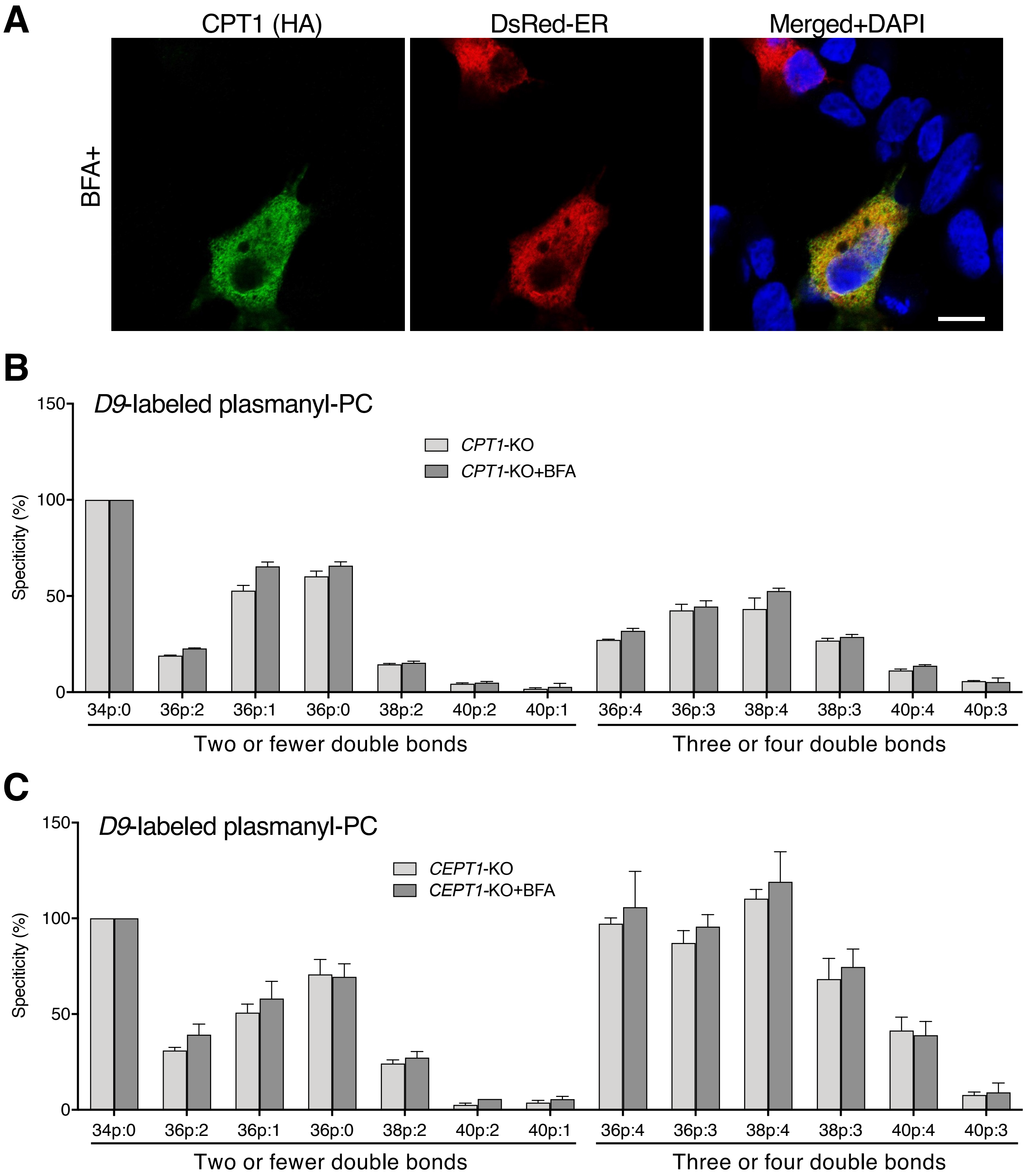

Supplemental Fig. S5. Effect of BFA on the specificity of the *de novo* biosynthesis of plasmanyl-PC molecular species in *CPT1*- or *CEPT1*-KO cells.

(A) Redistribution of HA-tagged CPT1 (green) to the ER after the BFA treatment (20 µg/ml for 4 hours). The ER was labeled with pDsRed-ER vector (red). Nuclei were stained with DAPI (blue). Bars indicate 10 µm. After pre-treatment with or without BFA for 2 hours, *CPT1*-KO (B) or *CEPT1*-KO (C) cells were incubated with *d9*-labeled choline for 2 hours in the presence or absence of BFA. After extraction, the levels of *d9*-labeled plasmanyl-PC molecular species were quantified using LC-MS/MS. The relative values of each plasmanyl-PC molecular species to 34p:0 were calculated and shown.

Supplemental Figure S5

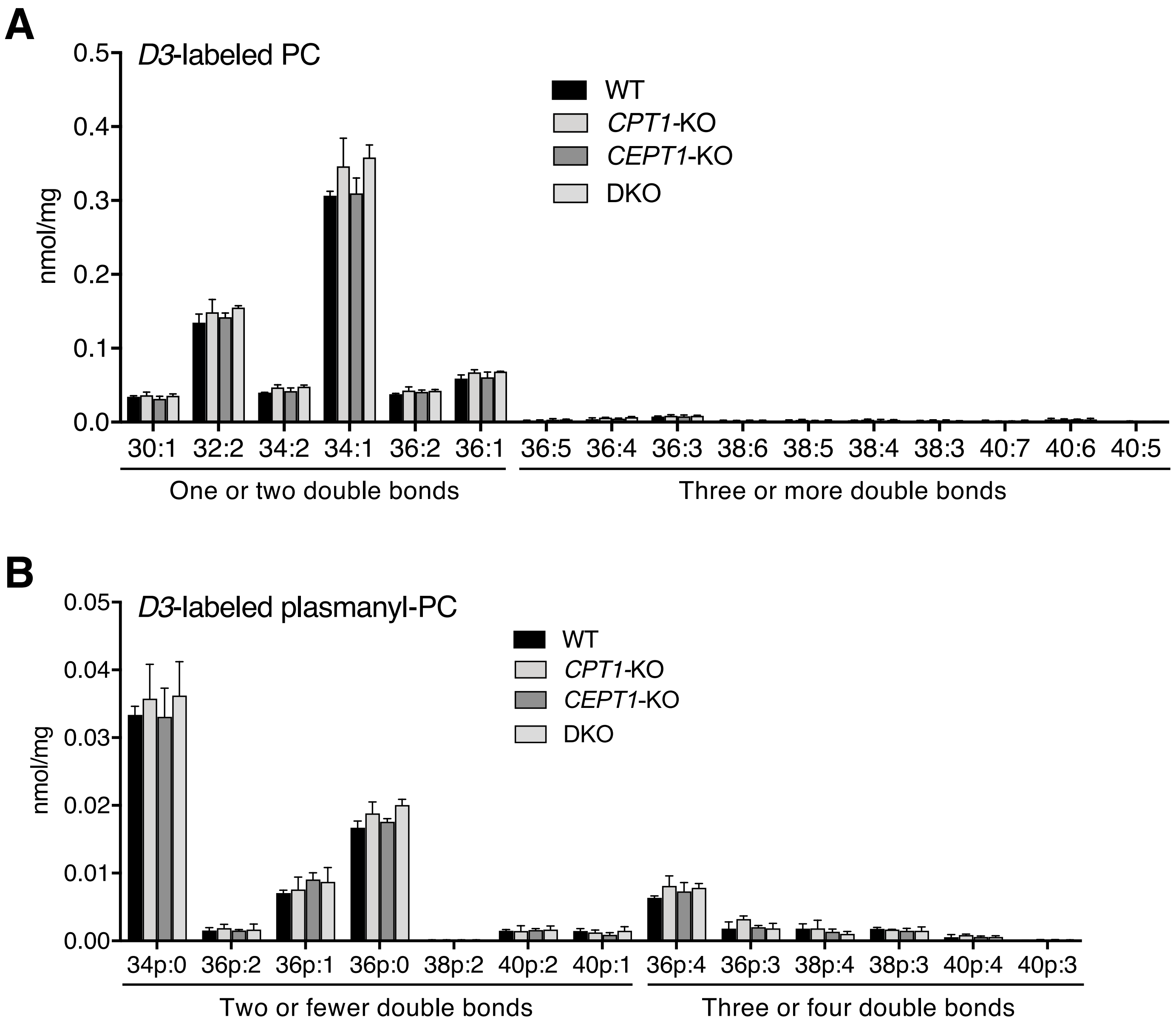

Supplemental Fig. S6. The *de novo* biosynthesis of PC and plasmanyl-PC from deuterium-labeled choline *via* the PE methylation pathway.

WT, *CPT1*-KO, *CEPT1*-KO, or DKO cells were incubated with *d9*-labeled choline for 2 hours. After extraction, the levels of *d3*-labeled PC (A) and plasmanyl-PC (B) molecular species were quantified using LC-MS/MS. The lipid amount was normalized by the amount of total cellular proteins. Data are means ± S.D. from three independent culture dishes.

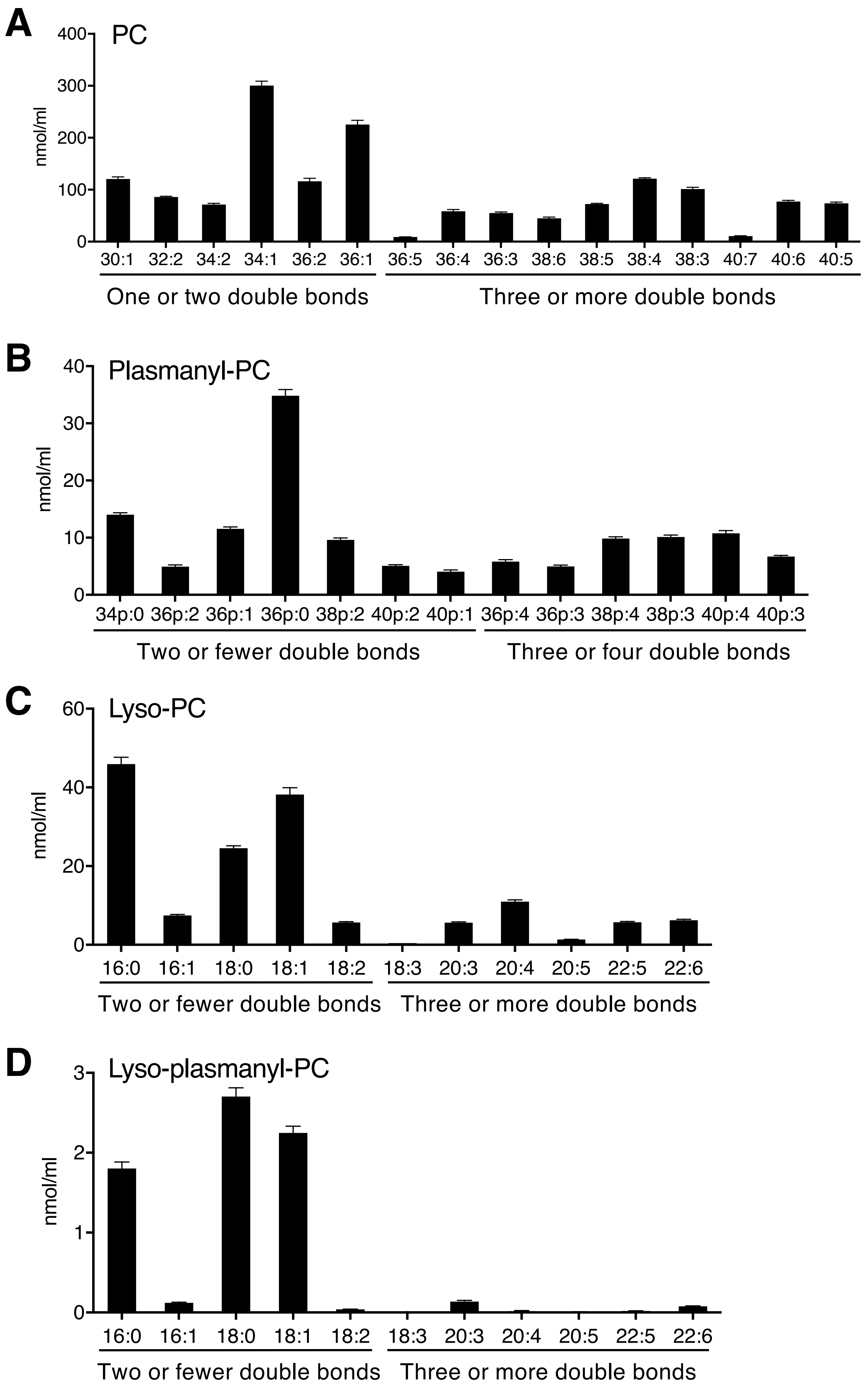

Supplemental Fig. S7. The levels of choline PLs in FBS.

The levels of PC (A), plasmanyl-PC (B), lyso-PC (C), and lyso-plasmanyl-PC (D) molecular species in FBS were quantified using LC-MS/MS. Data are means ± S.D. from three independent experiments.

Supplemental Figure S7

A

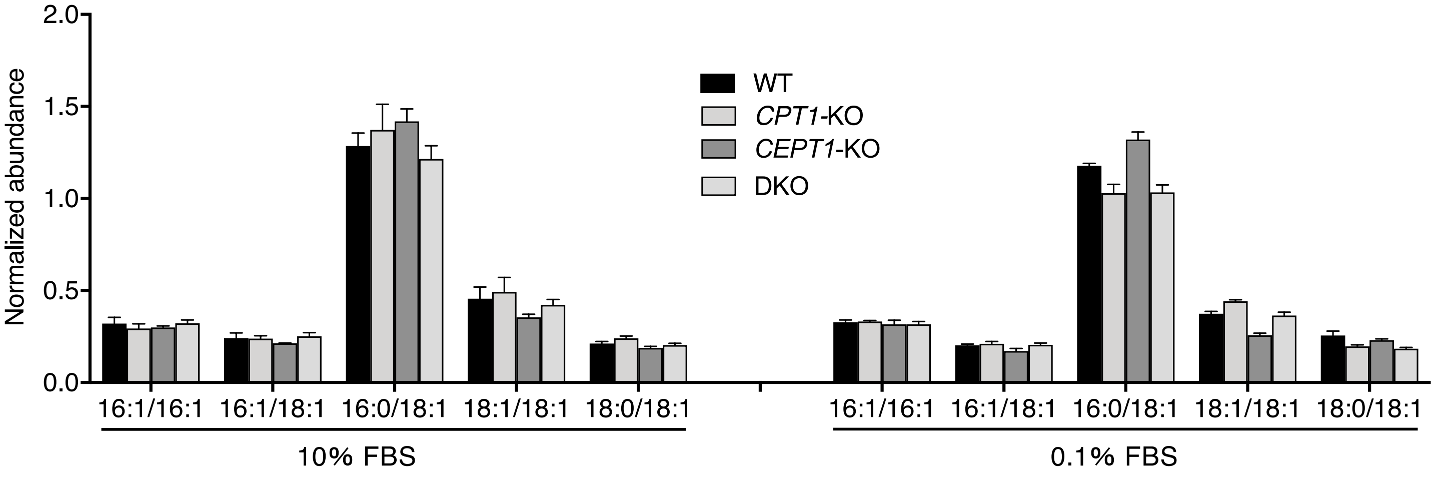

B

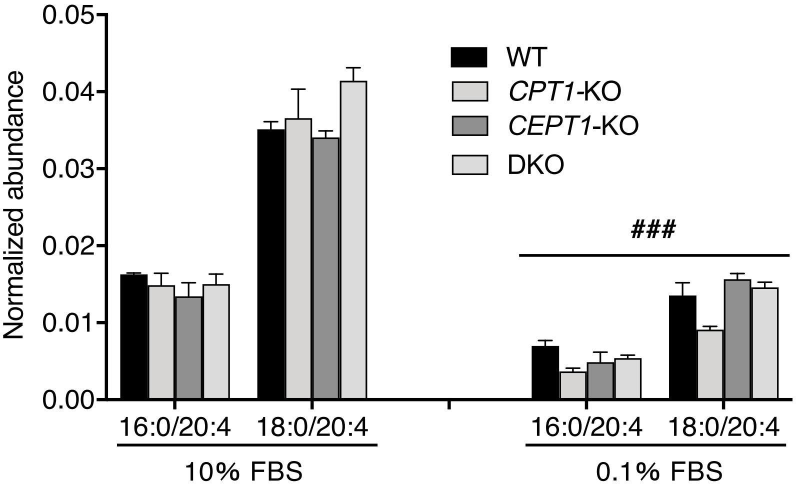

Supplemental Fig. S8. Amount of endogenous DAG molecular species in WT, *CPT1*-KO, *CEPT1*-KO, and DKO cells cultured with 10% FBS or 0.1% FBS.

Cells were cultured in HE100 medium containing 10% FBS or 0.1% FBS for 2 days. After lipids were extracted from the cells, the levels of DAG with palmitoleic acid or oleic acid (A) and arachidonic acid (B) were quantified using LC-MS/MS. Lipid levels were normalized by the amount of total cellular protein. Data are means ± S.D. from three independent culture dishes. ### indicates $P < 0.001$ as compared with 10% FBS. Statistical significance was determined using one-way ANOVA with Dunnett's post hoc test.

Supplemental Figure S8
